# Environmental Drivers of Vibrio cholerae Abundances in Mobile Bay, Alabama

**DOI:** 10.1128/spectrum.01733-22

**Published:** 2023-01-24

**Authors:** Elizabeth Leard, Ruth H. Carmichael, Alice C. Ortmann, Jessica L. Jones

**Affiliations:** a Food and Drug Administration, Division of Seafood Science and Technology, Gulf Coast Seafood Laboratory, Dauphin Island, Alabama, USA; b Department of Marine Sciences, University of South Alabama, Mobile, Alabama, USA; c University Programs, Dauphin Island Sea Lab, Dauphin Island, Alabama, USA; University of Maryland—Eastern Shore

**Keywords:** *V. cholerae* non-O1/O139, *ompW*, Mobile Bay, seasonality, NOVC

## Abstract

Vibrio cholerae is the etiological agent of the illness cholera. However, there are non-O1/non-O139 V. cholerae (NOVC) strains that generally lack the toxin gene (*ctx*) and colonization factors that cause cholera. These NOVC strains are autochthonous members of estuarine environments and a significant cause of seafood-borne gastroenteritis in the United States. The objective of this study was to identify environmental parameters that correlate with NOVC prevalence in oysters, water, and sediment at three ecologically diverse locations in Mobile Bay, AL, including Dog River (DR), Fowl River (FR), and Cedar Point (CP). Oyster, water, and sediment samples were collected twice a month when conditions were favorable for NOVC growth and once a month when they were not. A most probable number (MPN)/real-time PCR assay was used to determine NOVC abundances. Environmental parameters were measured during sampling to determine their relationship, if any, with NOVC at each site. NOVC abundances in oysters at DR, FR, and CP were 0.87, 0.87, and −0.13 log MPN/g, respectively. In water, the median NOVC levels at DR, FR, and CP were 1.18, −0.13, and −0.82 log MPN/mL, and in sediment, the levels were 1.48, 1.87, and −0.03 log MPN/g, respectively. Correlations of NOVC abundances in oyster, water, and sediment samples with environmental parameters were largely site specific. For example, the levels of NOVC in oysters at DR had a positive correlation with temperature but a negative correlation with dissolved oxygen (DO) and nutrient concentrations, NO_2_^−^, NO_3_^−^, dissolved inorganic nitrogen (DIN), total dissolved nitrogen (TDN), and dissolved inorganic phosphorus (DIP). At FR, however, the levels of NOVC in oysters displayed only a negative correlation with NO_2_^−^. When grouping NOVC abundances by temperature, the main driving factor for prevalence, additional correlations with salinity, total cell counts, dissolved organic nitrogen (DON), and dissolved organic carbon (DOC) became evident regardless of the site.

**IMPORTANCE** NOVC can cause gastrointestinal illness in humans, which typically occurs after the consumption of raw or undercooked seafood. Incidence rates of NOVC gastroenteritis have increased during the past decade. In this study, NOVC was enumerated from oysters, sediment, and water collected at three sites in Mobile Bay, with environmental parameters measured concurrently over the course of a year, to identify potential environmental drivers of NOVC abundances. The data from this study, from an area lacking in V. cholerae research, provide a useful baseline for risk analysis of V. cholerae infections. Defining correlations between NOVC and environmental attributes at different sites and temperatures within a dynamic system such as Mobile Bay provides valuable data to better understand the occurrence and proliferation of V. cholerae in the environment.

## INTRODUCTION

Vibrio cholerae is a pathogenic bacterium that causes the disease cholera by producing cholera toxin (CT) when colonizing the intestines of infected individuals. CT is responsible for the profuse, rice-water diarrhea characteristic of a cholera infection. Thin filamentous appendages on the surface of V. cholerae known as toxin-coregulated pili (TCP) facilitate colonization of the small intestine. Both major virulence factors are encoded on mobile genetic elements integrated into the chromosome of toxigenic V. cholerae O1 and O139 strains (1). With over 200 serogroups, however, most V. cholerae strains do not possess these major virulence factors and are generally designated non-O1/non-O139 V. cholerae (NOVC) (2).

NOVC strains are autochthonous members of estuarine environments worldwide, and while not associated with pandemic cholera outbreaks caused by the O1 and O139 serogroups, they can cause gastrointestinal illnesses in humans, typically after the consumption of raw or undercooked seafood (3). Virulence factors contributing to the pathogenicity of NOVC typically include the *rtx* (encoding repeat toxins), *toxR* (toxin regulatory gene), *hly* (encoding hemolysins), and *omp* (outer membrane proteins specific for V. cholerae) genes and a type III secretion system (T3SS) (4, 5). The role of these virulence factors is not well understood, but one or more have been isolated from patients suffering from severe diarrhea after consuming seafood (6, 7).

Sporadic outbreaks attributed to various NOVC strains are typically genetically diverse, while some strains, including the O141 strain, have been isolated from geographically distinct regions, including the United States, Spain, Taiwan, and India (8). Highlighting the importance of effective NOVC surveillance are the potential epidemiological links between clinical and environmental strains as well as the evolutionary relationships among isolated V. cholerae strains that are observed when using next-generation sequencing techniques (9, 10).

Recent studies have found that NOVC is a significant contributor to gastroenteritis infections and, to a lesser extent, otitis and bacteremia in both developed and developing countries globally (11–13). In the United States, NOVC was the third most commonly reported group of *Vibrio* bacteria, resulting in approximately 100 cases of NOVC infection each year (3). During a 1991 Mobile Bay, AL, environmental study, NOVC occurred in 26% and 14% of oysters sampled at warm (>20°C) and cold (<20°C) water temperatures, respectively (14). Because V. cholerae is an autochthonous member of Mobile Bay, AL (15), an increase in the threat of gastrointestinal illness acquired from the consumption of raw oysters remains a potential concern under conditions advantageous to V. cholerae proliferation.

At 1.13 × 10^5^ km^2^, Mobile Bay, on the Gulf of Mexico coast, is the sixth largest watershed in the United States (16). Mobile Bay is approximately 51.5 km long and 37 km wide at its widest point, with an average depth of about 3 m. An estimated 4.85 million metric tons of sediment enter the estuary annually, which, along with dissolved oxygen (DO) concentrations and temperature, can affect the nutrient content in the bay (17, 18). There is an average annual temperature range from nearly 10°C to over 30°C, a salinity gradient ranging from 0.8 ppt to 32 ppt, and a pH range from 5 to 9 (19–21). These environmental conditions in Mobile Bay are conducive for V. cholerae (14, 22), yet the site has a limited history of V. cholerae studies.

The objective of this study was to determine the effects of seasonal temperature variations and environmental attributes on V. cholerae abundances. This was done by collecting oysters, sediment, and water from three ecologically diverse locations around Mobile Bay throughout a calendar year. Being the first study of V. cholerae in the Mobile Bay area in more than a decade and the first to focus on NOVC, these data will further our understanding of environmental drivers of V. cholerae in a northern Gulf of Mexico estuarine system and provide baseline data for risk analysis of V. cholerae-associated illnesses.

## RESULTS

### V. cholerae abundances among sampling sites.

**FIG 1:**
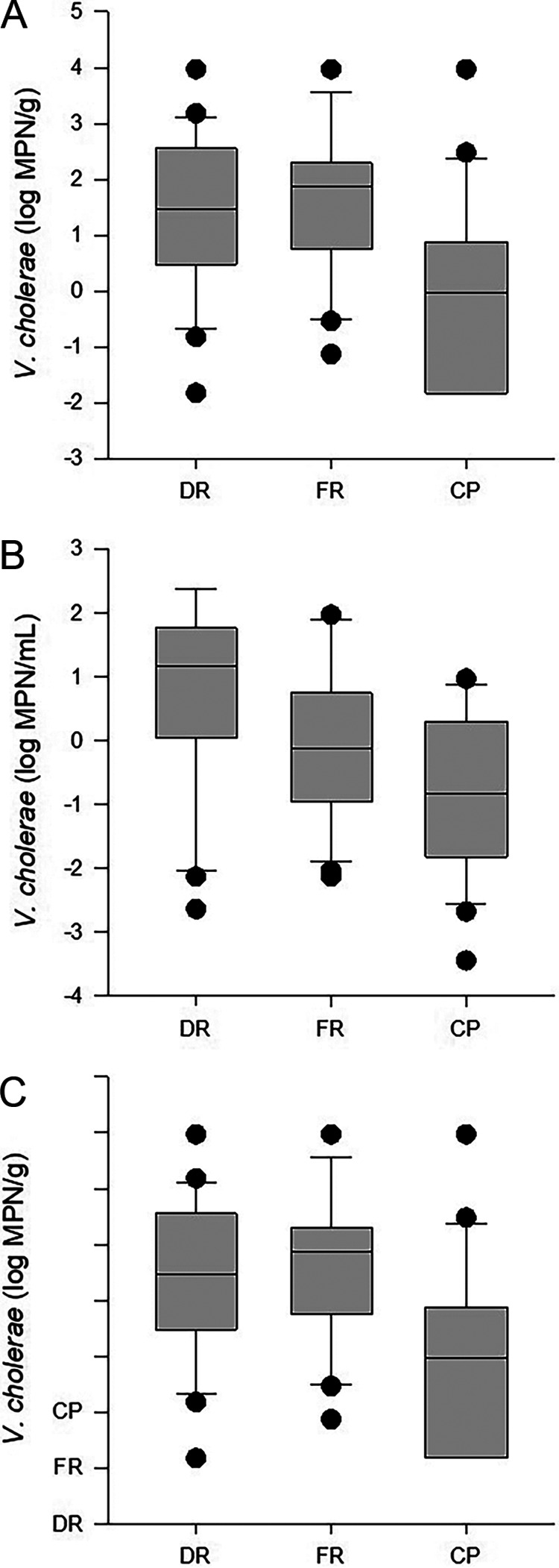
V. cholerae abundances in oyster (A), water (B), and sediment (C) samples at Dog River (DR), Fowl River (FR), and Cedar Point (CP). The lower boundary of each box indicates the 25th percentile, the line within the box marks the median, and the upper boundary of the box indicates the 75th percentile. Whiskers above and below the box indicate the 90th and 10th percentiles.

The median abundances of V. cholerae in oyster samples did not differ significantly (*P* = 0.16) among the Dog River (DR) (0.87 log most probable number [MPN]/g), Fowl River (FR) (0.87 log MPN/g), and Cedar Point (CP) (−0.13 log MPN/g) sites (Fig. 1A). In water and sediment (Fig. 1), however, the abundances of V. cholerae differed significantly among sites (*P* < 0.001 and *P* < 0.002, respectively). The median level of V. cholerae in water at DR (1.18 log MPN/mL) was higher than that at CP (−0.82 log MPN/mL) (*P* < 0.05), while V. cholerae abundances in water at FR (−0.131 log MPN/mL) were not significantly different from those at DR or CP. In sediment, the abundances of V. cholerae at DR and FR were 1.48 and 1.87 log MPN/g, respectively (Fig. 1C), both of which were significantly higher than the median value in CP sediment, −0.03 log MPN/g (*P* < 0.05).

The mean environmental parameters (Table 1) exhibited certain significant differences among sites. Salinity was significantly different between CP and FR (*P* = 0.02) and between CP and DR (*P* = 0.002). NO_2_^−^ and chlorophyll *a* (Chl *a*) were significantly different between CP and DR (*P* = 0.02 and *P* = 0.04), and total dissolved nitrogen (TDN) and particulate carbon (PC) were significantly different between CP and FR (*P* = 0.04 and *P* = 0.03).

**TABLE 1:** Yearly values of environmental parameters in Alabama waters that were correlated with V. cholerae abundances at each sampling site

Site^*a*^	Mean temp (°C) ± SE	Mean salinity (ppt) ± SE	Mean DO concn (mg/mL) ± SE	Mean pH ± SE	Mean total cell count (log_10_) ± SE		Mean NO_2_^−^ concn (μM) ± SE	Mean NO_3_^−^ concn (μM) ± SE	Mean TDN concn (μM) ± SE	Mean DIP concn (μM) ± SE	Mean DOC (ppm) ± SE
					Sediment	Water					
DR	22.5 ± 1.52	7.11 ± 0.90	7.57 ± 0.45	7.32 ± 0.18	5.14 ± 0.12	4.71 ± 0.31	0.16 ± 0.05	2.49 ± 1.0	36.22 ± 2.29	0.20 ± 0.03	4.07 ± 0.13
FR	22.64 ± 1.51	9.61 ± 1.20	7.58 ± 0.48	7.35 ± 0.19	5.02 ± 0.16	4.72 ± 0.30	0.12 ± 0.05	1.65 ± 0.85	37.04 ± 2.12	0.22 ± 0.03	4.38 ± 0.35
CP	22.81 ± 1.45	14.49 ± 1.57	7.79 ± 0.43	7.45 ± 0.20	5.15 ± 0.15	4.79 ± 0.36	0.07 ± 0.04	1.96 ± 0.90	31.12 ± 1.85	0.91 ± 0.65	3.92 ± 0.15

a DR, Dog River; FR, Fowl River; CP, Cedar Point.

### Vibrio cholerae abundances within sites and environmental correlates.

At DR, V. cholerae levels ranged from −1.82 to 5.46 log MPN/g in oysters, −2.64 to 2.38 log MPN/mL in water, and 1.82 to 3.97 log MPN/g in sediment (Fig. 1A to C). The abundances of V. cholerae in oysters were positively correlated with temperature and negatively correlated with DO, NO_2_^−^, NO_3_^−^, dissolved inorganic nitrogen (DIN), and TDN. V. cholerae levels in water at DR were similarly correlated with temperature and DO, which ranged from 3.38 to 11.02 mg/L, and with V. cholerae levels in oysters (Table 2). The concentrations of NO_2_^−^ and NO_3_^−^ ranged from below the limit of detection (LOD) to 0.8 μM and from below the LOD to 13.76 μM, respectively, while the concentration of DIN ranged from 0.36 to 16.96 μM, and that of TDN ranged from 22.6 to 55.6 μM. Temperatures at DR ranged from 10°C to 32°C.

**TABLE 2:** Environmental parameters with correlations with V. cholerae abundances by sampling site^*a*^

Site	Sample type	Environmental parameter (median value)	*R* value	*P* value	95% CI (lower, upper)
DR	Oyster	Temp (23.3°C)	0.75	<0.001	0.449, 0.898
		DO (8.23 mg/mL)	−0.67	0.001	−0.862, −0.310
		NO_2_^−^ (0.01 μM)	−0.59	0.007	−0.824, −0.186
		NO_3_^−^ (0.04 μM)	−0.67	0.001	−0.862, −0.310
		DIN (1.32 μM)	−0.66	0.002	−0.857, −0.294
		TDN (32.47 μM)	−0.48	0.04	−0.774, −0.017
	Water	Temp (23.3°C)	0.68	0.001	0.327, 0.867
		Abundance in oysters (1.37 log MPN/g)	0.61	0.004	0.229, 0.829
		DO (8.23 mg/mL)	−0.63	0.004	−0.843, −0.246

FR	Oyster	NO_2_^−^ (0.01 μM)	−0.49	0.03	−0.766, −0.061
	Water	DIP (0.17 μM)	−0.54	0.02	−0.793, −0.128
	Sediment	DO (7.36 mg/mL)	0.53	0.02	0.114, 0.788

CP	Oyster	Abundance in water (−0.83 log MPN/mL)	0.46	0.04	0.035, 0.744

a CI, confidence interval.

At FR, the median V. cholerae levels in oysters, water, and sediment ranged from −1.82 to 3.63 log MPN/g, −2.13 to 1.96 log MPN/mL, and −1.13 to 3.97 log MPN/g, respectively (Fig. 1A to C). V. cholerae abundances in oysters were negatively correlated with NO_2_^−^, while V. cholerae abundances in water were negatively correlated with dissolved inorganic phosphorus (DIP), and the levels of V. cholerae in sediment were positively correlated with DO (Table 2). The NO_2_^−^ concentration ranged from below the limit of detection (<0.02 μM) (23) to 0.8 μM, DIP had a concentration range from 0.08 to 0.68 μM, and DO had a concentration range from 2.78 to 11.65 mg/L.

At CP, V. cholerae levels in oysters, water, and sediment ranged from −1.82 to 2.85 log MPN/g, −3.44 to 0.97 log MPN/mL, and −1.82 to 3.97 log MPN/g, respectively (Fig. 1A to C). V. cholerae abundances in oysters were positively correlated with values in water but not environmental attributes (Table 2).

### V. cholerae abundances during warm and cold periods.

By stratifying V. cholerae abundances and environmental parameters into warm and cold periods, the relationship between V. cholerae and the environment is observed based on seasonality.

**FIG 2:**
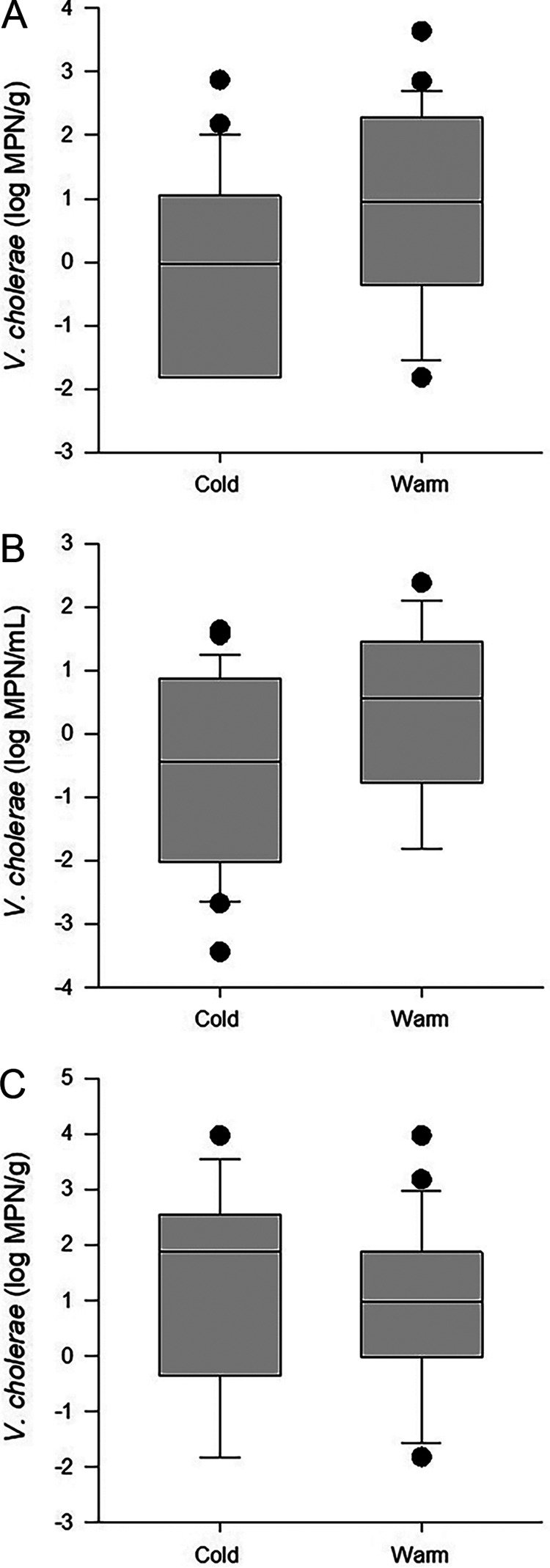
V. cholerae abundances in oyster (A), water (B), and sediment (C) samples stratified by warm and cold water temperatures (defined as temperatures above and below 20°C, respectively). The boundaries of each box indicate the 25th percentile, the median, and the 75th percentile, with whiskers indicating the 10th and 90th percentiles.

In oysters, V. cholerae abundances (Fig. 2A) were significantly different between warm- and cold-weather collections, with the median value of 0.96 log MPN/g during warm temperatures being higher than the median value of 0.26 log MPN/g during cooler periods (*P* < 0.04). The abundances of V. cholerae in water (*P* = 0.08) (Fig. 2B) and sediments (*P* = 0.80) (Fig. 2C) did not differ significantly between warm (≥20°C) and cold (<20°C) periods. The median values for V. cholerae in water were 0.55 and −0.28 log MPN/mL during warm and cold periods, respectively, and those in sediments were 0.97 and 1.04 log MPN/g, respectively.

At warm temperatures, a positive correlation was observed between V. cholerae abundances in oysters (which ranged from −1.82 to 3.63 log MPN/g) and abundances in water (−1.82 to 2.38 log MPN/mL) and sediment (−1.82 to 3.97 log MPN/g) (Fig. 2). V. cholerae abundances in water during periods of warmer temperatures were negatively correlated with salinity and positively correlated with V. cholerae levels in sediment, TDN, and dissolved organic nitrogen (DON) (Table 3). V. cholerae in sediment was also negatively correlated with salinity. Salinity ranged from 2.1 to 22.5 ppt, the TDN concentration ranged from 21.5 to 47.3 μM, and the DON concentration ranged from 20.8 to 47.3 μM.

**TABLE 3:** Environmental parameters significantly correlated with V. cholerae abundances during cold and warm sampling periods (defined as temperatures of ≥20°C and <20°C, respectively)

Sampling period	Sample type	Environmental parameter (median value)	*R* value	*P* value	95% CI (lower, upper)
Warm	Oyster	Abundance in sediment (0.97 log MPN/g)	0.56	0.004	0.284, 0.751
	Water	Abundance in oysters (0.96 log MPN/g)	0.46	0.005	0.155, 0.685
		Abundance in sediment (0.97 log MPN/g)	0.54	0.001	0.257, 0.738
		DON (30.65 μM)	0.52	0.002	0.221, 0.729
		TDN (32.37 μM)	0.52	0.002	0.221, 0.729
		Salinity (11.1 ppt)	−0.54	0.001	−0.738, −0.257
	Sediment	Salinity (11.1 ppt)	−0.50	0.002	−0.712, −0.205

Cold	Water	Temp (15.3°C)	0.49	0.02	0.108, 0.755
		DOC (4.03 ppm)	0.42	0.04	0.019, 0.704
		Total cell count in water (6.26 log_10_ cells)	0.54	0.02	0.098, 0.804
		Salinity (5.5 ppt)	−0.59	0.003	−0.802, −0.245

The ranges for V. cholerae levels in water, sediment, and oysters during periods of cold water temperatures (9.6°C to 19.0°C) were −3.44 to 1.63 log MPN/mL, −1.82 to 3.97 log MPN/g, and −1.82 to 2.87 log MPN/g, respectively (Fig. 2). Positive correlations were observed between the abundances of V. cholerae in water and the temperature, DOC concentration, and total bacterial cell counts from water, but a negative correlation was observed for salinity, which ranged from 0.8 to 23.3 ppt. DOC ranged from 2.58 to 9.82 ppm (Table 3).

## DISCUSSION

Due to the increase in NOVC-related gastroenteritis during the past decade and a general lack of data on V. cholerae ecology in the northern Gulf of Mexico, we examined V. cholerae abundances at three sites around Mobile Bay, AL, for 1 year. Environmental variables were measured concurrently to elucidate the relationship between V. cholerae and its environment. V. cholerae was present in water, sediment, and oysters at all sites throughout the year, and correlations with environmental parameters varied based on the collection site and warm versus cold collection periods (Fig. 2). Because of the ability to thrive in dynamic environments, V. cholerae flourishes in many different types of estuaries worldwide (24–26), including those like Mobile Bay that are characterized by wide ranges of temperatures, salinities, pHs, and other environmental variables favorable to V. cholerae proliferation (27).

**FIG 3:**
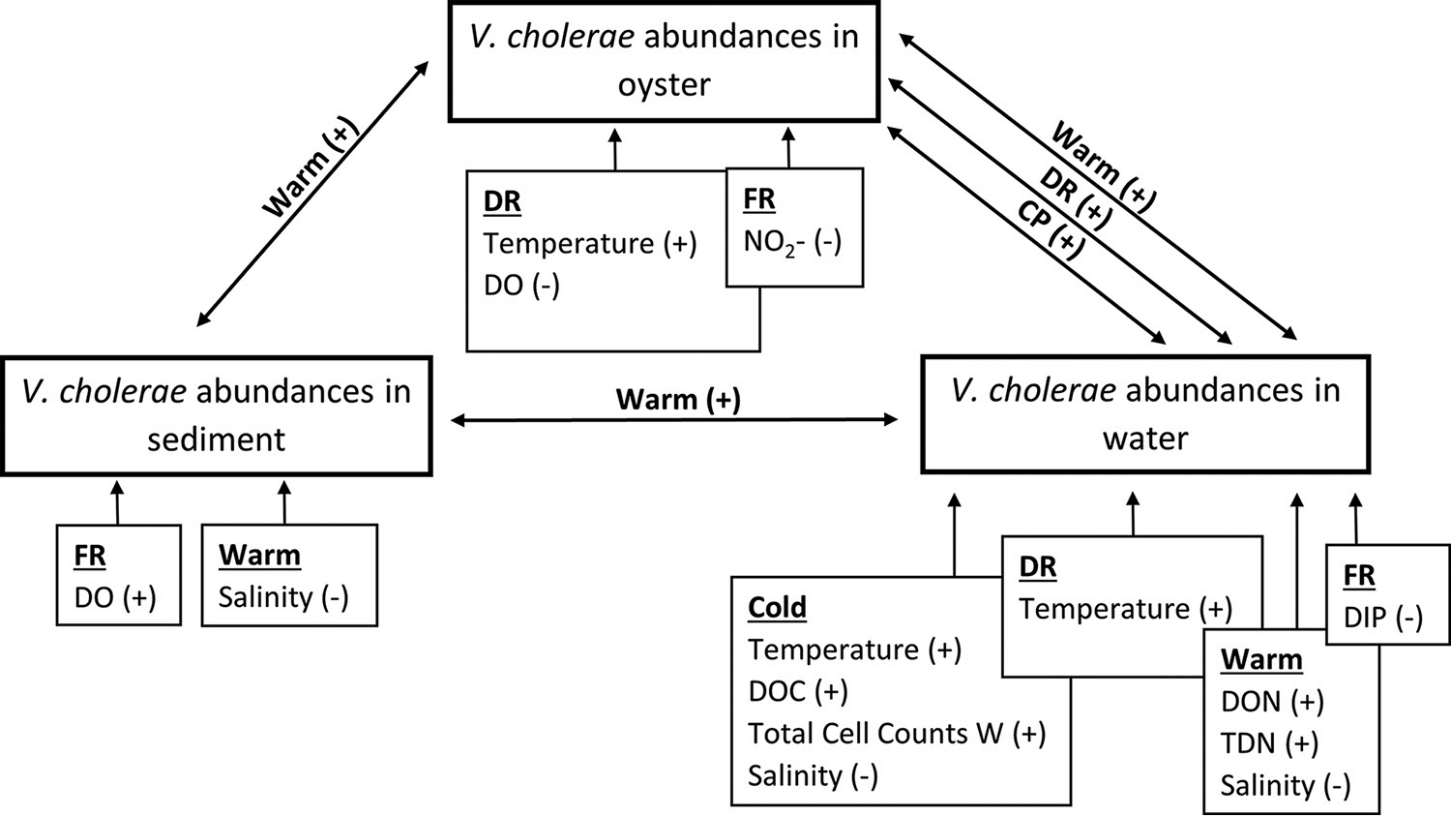
Schematic of the interactions between V. cholerae abundances (boxes with thick dark borders) and correlating environmental parameters (boxes with arrows pointing to V. cholerae abundances) measured during this study. Negative (−) or positive (55) correlations are shown for each site (DR, FR, and CP) and sampling period (cold or warm).

The relationships between V. cholerae abundances and environmental attributes (by site or sampling period) were complex (Fig. 3). While temperature and salinity were significantly correlated with V. cholerae abundances in Mobile Bay, this relationship was not evident at every site, among every sample type, or during all sampling periods. This site specificity was also evident between V. cholerae and the other environmental attributes tested. Additionally, V. cholerae abundance was related to biological factors such as total cell counts and V. cholerae abundances in the other sample types. *Vibrio* species, including V. cholerae, are frequently positively correlated with other organisms, especially copepods, indicating a commensal relationship (28–30). This relationship that V. cholerae has with the surrounding community may explain the positive correlation with total cell counts as well as V. cholerae abundances in each of the sample types during cold and warm sampling periods. Because of the inverse relationship between DO and temperature, a strong negative correlation between V. cholerae levels in water and oysters at DR is not unexpected (28, 31). However, V. cholerae abundances in sediment at FR exhibited a positive correlation with DO. These increases in abundance and DO together can happen during periods of cold temperatures, which supports the idea that *Vibrio* may overwinter in sediments (32). The abundant supply of nutrients available to V. cholerae may explain the positive correlation between V. cholerae levels and nutrients in water during warm- and cold-weather sampling. Conversely, there was a negative correlation between nutrients and V. cholerae levels in oysters, which gives a more comprehensive overview of the environment than the cursory glance obtained from a water sample (33). Nutrient sources in the environment are not distributed evenly and typically occur as microscale patches (34). More research is needed to resolve these complex, site-specific relationships.

This study supports previous findings of the site specificities of correlations between V. cholerae and environmental parameters among multiple sites within a single estuary. In a study in France, oysters, sediment, and water were sampled from three lagoons on the Mediterranean coast where V. cholerae levels ranged from 20 to 40 MPN/L (1.30 to 1.60 log MPN/L) and were found only in water and sediment samples during the warm season in two of the lagoons when all three were within 40 km of each other (24). While V. cholerae is highly adaptive to its environment, likely causing abundances to be site specific, seasonality is still considered the leading environmental driver in risk assessment, with an increasing focus on the relationship between V. cholerae and phytoplankton in prediction efforts (35). During a study at Wassaw Sound and Sapelo Sound along the coast of Georgia in the United States, V. cholerae levels had a distinct association with seasons and taxonomic shifts in plankton species (34, 35).

This study provides a baseline understanding of the relationship between V. cholerae and its environment in the Mobile Bay estuary system. This information can form the foundation for risk assessment efforts for V. cholerae infections from oysters or recreational exposure along the U.S. Gulf of Mexico coast. While much additional information is needed for a full risk assessment of V. cholerae in water or oysters at the level of those done for Vibrio parahaemolyticus in seafood, V. vulnificus in oysters, or V. cholerae O1 and O139 in shrimp (36–38), the identified relationships between V. cholerae and certain environmental factors in this study provide a solid basis for future work.

## MATERIALS AND METHODS

### Site selection.

**FIG 4:**
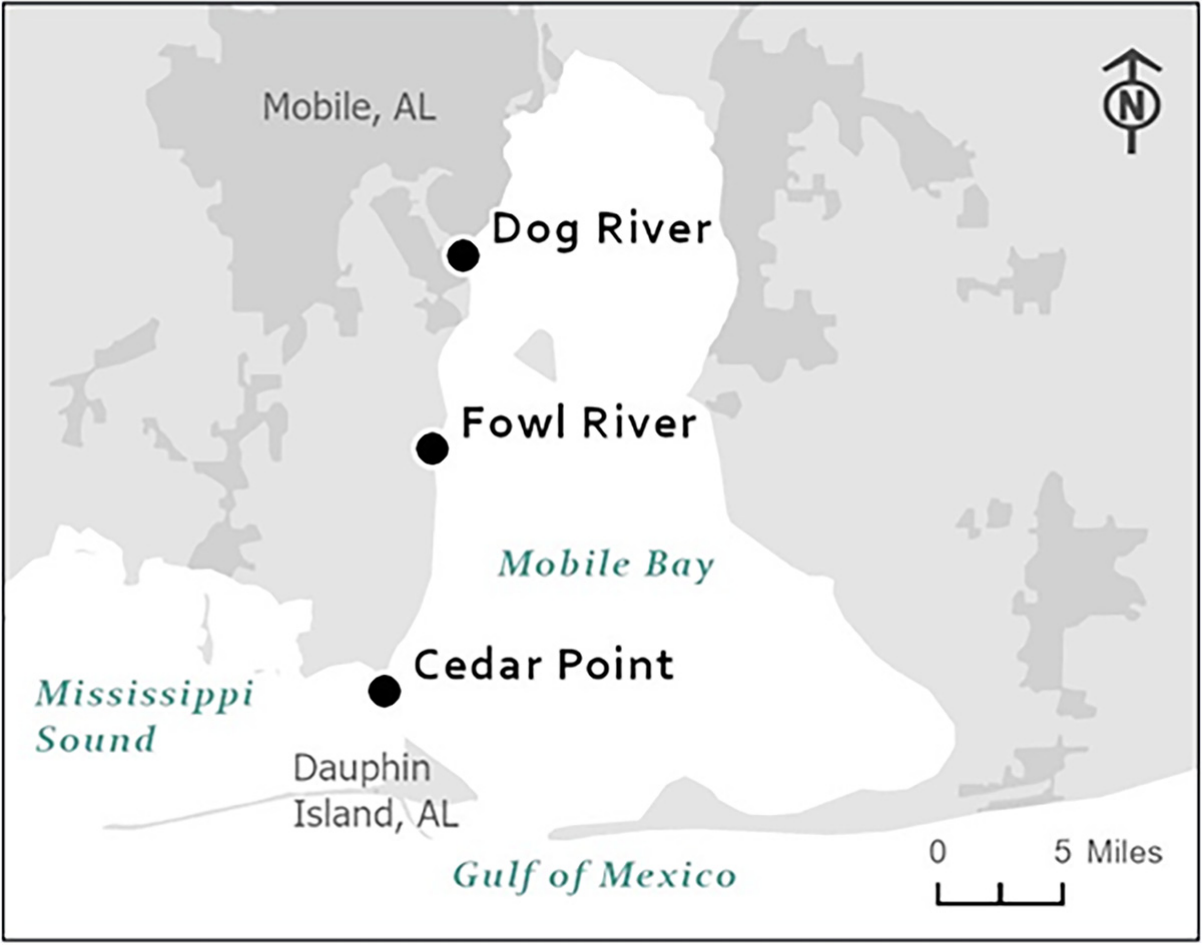
Vibrio cholerae sampling sites at the mouth of Dog River, Fowl River, and Cedar Point near Mobile Bay, AL. (Image courtesy of the FDA Geographic Information Systems and the U.S. Census Bureau, 2020.)

Sampling was conducted at three locations along a salinity gradient in the Mobile Bay-Mississippi Sound system (Fig. 4). The northernmost site (DR) is located at the mouth of Dog River, a 13-km-long, tidally influenced shallow river (39). The Fowl River (FR) site is located near Fowl River, a 23.2-km-long, brackish river originating near Theodore, AL (39). The Cedar Point (CP) site is in the Mississippi Sound, north of Dauphin Island, a barrier island located 3 mi south of the mouth of Mobile Bay in the Gulf of Mexico.

### Sample collection.

Sampling was conducted twice a month from May 2012 to May 2013 when water temperatures were within the optimal range for V. cholerae growth (20°C to 45°C) (40) and was decreased to once a month when water temperatures were <20°C. A sterile 5-L container was used to collect water at each site by immersing the container in the upper 0.5 m of the water column. Approximately 20 g of sediment was then collected with a stainless steel 3-L sediment corer based on the Van Veen design and transferred to a 110-mL polypropylene sterile collection container with a lid (Corning, Pittsburgh, PA). Finally, 12 oysters were collected by dredging at each site on each collection date. All samples were stored in a cooler on bagged ice and analyzed upon return to the laboratory, which was no longer than 4 h after collection.

Dissolved oxygen (DO), salinity, and surface water temperature were determined during each collection in the field with a YSI handheld meter (model 85; YSI Incorporated, Yellow Springs, OH). pH and turbidity were measured in the laboratory using a Mettler Toledo (Columbus, OH) EL20 pH meter and a La Motte (Chestertown, MD) 2020 turbidimeter, respectively.

### Sample processing.

A most probable number (MPN)/real-time PCR assay was used to determine the abundances of V. cholerae in oyster, water, and sediment samples. To obtain a representative estimate of the abundance of V. cholerae in the oyster community, 12 oysters from each site were opened aseptically and homogenized using a sterile blender. For oysters and sediment, 90 mL of alkaline peptone water (APW) (41) was inoculated with 10 g of the oyster homogenate or sediment, and 9 mL of APW was inoculated with 1 g of the oyster homogenate or sediment. Tenfold serial dilutions of the oyster homogenate and sediment were made in phosphate-buffered saline (PBS) (23) through a 10^−4^ dilution, and 1 mL of each dilution was inoculated into 9 mL APW. For water samples, 100 mL of a 10× APW stock was inoculated with 1 L of water, 10 mL of 10× APW was inoculated with 100 mL of water, and 1 mL of 10× APW was inoculated with 10 mL of water. Tenfold serial dilutions of water were made in PBS through a 10^−4^ dilution, and 1 mL of each dilution was inoculated into 9 mL of APW. All dilutions were inoculated in triplicate for a 3-tube MPN series, and APW tubes were incubated at 42°C ± 1°C overnight as recommended in the FDA *Bacteriological Analytical Manual* (41).

A 1-mL aliquot of the MPN overnight enrichment from each tube showing turbid growth was transferred to a microcentrifuge tube and heated at 100°C for 10 min. Sample aliquots were immediately cooled on ice or frozen at temperatures of below −15°C. If frozen, samples were thawed completely before use. Sample tubes were centrifuged at 8,000 relative centrifugal force (RCF) for 60 s, and the resulting supernatant was used in a real-time PCR assay to specifically detect V. cholerae (see Tables S1 and S2 in the supplemental material), targeting the *ompW* gene (7500 Fast; Applied Biosystems, Foster City, CA) (Table 4) with an internal amplification control (IAC), as previously described (15). The development of the V. cholerae real-time PCR assay is described in the supplemental material. Real-time PCR was conducted using an optimized 25-μL reaction mixture containing (final concentrations) Invitrogen Express supermix (2×), forward and reverse V. cholerae primers (0.25 μM each), a V. cholerae probe (0.1 μM), IAC forward and reverse primers (0.2 μM each), an IAC probe (0.1 μM), ROX reference dye (0.004 μM), an IAC template (1 μL of a diluted, purified plasmid), and a V. cholerae template (2 μL of the boiled enrichment). Amplification was conducted with an initial denaturation step (95°C) for 2 min and 40 cycles of denaturation (95°C for 1 min), annealing (60°C for 1 min), and elongation (72°C for 1 min). Default analysis settings were used, except that the threshold was set at 0.015 (Fig. S1). Detection of V. cholerae by real-time PCR was used qualitatively to determine positive/negative tubes in the MPN series.

**TABLE 4:** Sequences of real-time PCR primers and probes used in the assay

Primer or probe	Sequence (5′→3′)^*a*^	Reference
OmpW 98F	TTGCCTCGGTAGTACCTAATGACA	This study
OmpW 257R	TCACCACCAGAGGTAGAAATCTTATG	This study
OmpW probe	FAM-TGAAGTCCTCGCTGCTACGCCATTTTC-BHQ1	This study
IAC 46F	GACATCGATATGGGTGCCG	15
IAC 219R	GAGCCAAGTCAGATGATGGTACG	This study
IAC probe	CY5-TCTCATGCGTCTCCCTGGTGAATGTG-BHQ2	15

a FAM, 6-carboxyfluorescein; BHQ1, black hole quencher 1.

### Nutrient and chlorophyll analysis.

Total dissolved nitrogen (TDN) (micromolar) and dissolved inorganic nitrogen (NO_2_^−^, NO_3_^−^, and NH_4_^+^) were measured in water samples from each site on the Skalar San++ autoanalyzer, using glutamic acid as a standard (42). Water samples (60 mL) were filtered through Whatman glass fiber filters (25 mm, grade GF/F, 0.7 μm) that had been muffled for 4 h at 450°C. The dissolved organic nitrogen (DON) concentration (micromolar) was determined by subtracting DIN from TDN (43, 44). Dissolved organic carbon (DOC) (parts per million) was measured from water samples (10 mL) filtered through Whatman glass fiber filters (25 mm, grade GF/F, 0.7 μm) and analyzed on the TOC-5000 instrument, using potassium biphthalate as a standard (45). The filter was placed into a muffled 25-mL-capacity vial and frozen until particulate carbon analysis was performed.

Total dissolved phosphorous (TDP) (micromolar) and phosphate (PO_4_^3−^) (micromolar) were measured from 10 mL of filtered water sampled at each site. Samples were placed into muffled scintillation vials with 0.2 mL of 0.17 M MgSO_4_ and evaporated to dryness at 95°C. The residue was baked at 450°C to decompose organic phosphorus compounds. The dissolved organic phosphorous (DOP) concentration (micromolar) was calculated as the difference between TDP and PO_4_^3−^. Particulate organic phosphorous (POP) (micromolar) was measured from 100-mL filtered water samples. Filters were rinsed twice with 2-mL aliquots of 0.17 M Na_2_SO_4_, placed into muffled scintillation vials with 2 mL 0.017 M MgSO_4_, dried at 90°C, and stored in a drying oven at 40°C. All phosphorus-based analyses were performed on the Shimadzu UV-160 spectrophotometer at 885 nm, using potassium phosphate as a standard (46, 47). Total particulate carbon (PC) (micromolar) and particulate nitrogen (PN) (micromolar) were measured by high-temperature combustion using the Carlo-Erba NA 1500 CNS analyzer, with atropine as the standard (48, 49).

For the determination of chlorophyll *a* (Chl *a*) concentrations (per microgram), fluorescence was measured for 100-mL water samples. Each sample was filtered through a 25-mm Whatman glass fiber filter (grade GF/F, 0.7 μm), which was folded in half and submerged in 10 mL acetone (90%). The samples were then frozen for 24 h. The samples were then shaken vigorously and centrifuged for 10 min at 2,205 RCF. The fluorescence of the samples was measured using a Turner Designs TD-700 fluorometer calibrated with a chlorophyll *a* solid standard (50).

### Total cell counts.

A standard 4′,6-diamidino-2-phenylindole (DAPI) protocol was used to determine total cell counts in water and sediment. Water (4.9 mL) from each site was mixed with 0.1 mL glutaraldehyde and held at 2°C to 4°C for 15 min. A 0.2-μm polycarbonate filter was placed over a backing filter on a Millipore (Bedford, MA) model 1225 sampling manifold vacuum filtration cell harvester. Two milliliters of the sample and 50 μL of DAPI were placed onto the vacuum and allowed to stand for 2 min. The sample and stain were drawn through the filter, which was then removed from the vacuum and allowed to dry before affixing to a slide. Labeling conditions vary by cell type, and the protocol was modified for this application (51). To determine total cell counts in sediment, samples (1.5 g to 2 g) were mixed with 30 mL of water at 18.2 MΩ and 4 mL of 37% formaldehyde. Filtered sodium tetrapyrophosphate (0.02 mL) was then added to the samples, and the samples were shaken gently for 15 min to mix and then sonicated (60 W, 3 times for 20 s) (Fisher Scientific ultrasonic cleaner). After dilution to 1:2,000 with water at 18.2 MΩ, 2 mL of the sample was mixed with DAPI (0.01 μg mL^−1^), incubated for 2 min, and filtered through a black 0.2-μm-pore-size polycarbonate filter with a cellulose-nitrate backing filter. The filter was washed with 2 mL of 18.2-MΩ water, removing the unbound DAPI, and then dried in the dark. Filters were mounted onto a slide and frozen at −20°C until they were ready to be examined by epifluorescence microscopy (52, 53). The final bacterial density was determined using the equation bacteria/mL = membrane conversion factor × *ND*, where the membrane conversion factor is the filtration area/area of the micrometer field, *N* is the total number of bacteria counted/number of micrometer fields counted, and *D* (dilution factor) is the volume of the sample stained/total volume of the sample available.

### Statistical analysis.

Data were analyzed by location and warm or cold seasonal conditions. Warm conditions were defined as any sampling period when water temperatures were ≥20°C, while cold conditions were defined as periods with temperatures of <20°C. Enumeration of V. cholerae was done using standard MPN tables based on real-time PCR results (54). Half the limit of detection (LOD) was substituted for outcomes at nondetectable levels (<3.0 MPN/g or L), and data were then log_10_ transformed. Analysis of variance (ANOVA) with a Tukey *post hoc* test was used to determine significant differences among V. cholerae abundances in oysters, sediment, and water among sites. As the environment data were nonnormal, differences in environmental parameters among sites were evaluated using Kruskal-Wallis ANOVA with Tukey’s *post hoc* test. Significant differences between the abundances of V. cholerae during cold- and warm-water periods were analyzed using a Mann-Whitney-Wilcoxon test. Pearson correlation analysis was used to determine the relationship between V. cholerae abundances and environmental parameters when tested by site and seasonality. A significance level of a *P* value of <0.05 was set for all analyses for correlations between V. cholerae abundances and environmental parameters at different sites and temperatures. All analyses were conducted using SigmaPlot 12.5 (Systat Software, Inc., San Jose, CA).
