# Supplementary material for: Environmental Drivers of Vibrio cholerae Abundances in Mobile Bay, Alabama
Source: Microbiol Spectr. 2023 Jan 24;11(1):e01733-22. doi: 10.1128/spectrum.01733-22 (PMC9927273; doi:10.1128/spectrum.01733-22)
Supplement: Supplemental file 1 — Supplemental material. Download spectrum.01733-22-s0001.pdf, PDF file, 0.3 MB [file spectrum.01733-22-s0001.pdf]

Development of a real time PCR assay for the detection of the *Vibrio cholerae ompW* gene

Using design tools available from Integrated DNA Technologies (IDT; www.idtdna.com), forward and reverse primers were developed for this study targeting a 304 bp section, specific to *Vibrio cholerae*, *omp*W gene, which codes for the outer membrane protein. A sequence-specific oligonucleotide TaqMan® probe was developed using Primer Express 3.0. A BLAST alignment was run to ensure the selected primers and probe were unique to the desired target sequence. An internal amplification control (IAC) developed by Blackstone et al. (2007) was included in the PCR assay to eliminate the reporting of false negative results [1]. The IAC reverse primer was optimized for this study to improve amplification according to the cycling parameters.

A total of 47 *V. cholerae* isolates (Table S1) along with 27 non-*V. cholerae* and non-vibrio isolates (Table S2), previously characterized by standard biochemical procedures, were tested to evaluate the efficacy of *ompW* primers and probe for the identification of *V. cholerae*. Isolates are part of the FDA Gulf Coast Seafood Laboratory (GCSL) collection with clinical and environmental isolates obtained locally, across the U.S., and internationally. All isolates gave expected results, with the exception of one. Isolate Vm3, which was previously characterized as *Vibrio mimicus,* was identified as *V. cholerae* using the PCR assay and was confirmed to be *V. cholerae* after further biochemical screening was performed.

A mid-exponential phase of *V. cholerae,* clinical strain VRL 1984, was serially diluted tenfold, with the highest dilution at 10^{-7}, in sterile phosphate-buffered saline (PBS). Duplicate aliquots of each dilution were spread plated onto T_1N_1 media, and a 1 mL aliquot of each dilution was boiled for use as the real-time PCR template. A 1:100 oyster enrichment matrix, negative for

V. cholerae, was prepared to test for inhibition. A standard curve was generated using 1 μL of each serial dilution and 1 μL boiled oyster matrix as real-time PCR template in triplicate (Fig S1). This assay has a sensitivity of 0.8 colony forming units (CFU) per reaction with an efficiency of 94%.

Table S1 Results of inclusivity testing for *Vibrio cholerae* isolates using the primers and probes for the *omp*W gene.

Vibrio cholerae				
**FDA GCSL Isolate Number**	**Isolate Name**	**PCR results**	**Source**	**Origin**
Vc1	CDC 3569-03	+	US	Clinical
Vc2	C-6706	+	Peru	Clinical
Vc3	CDC F851	+	US	Clinical
Vc4	SJ 21	+	US - CA	Environmental
Vc5	CDC 3541-98	+	US	Clinical
Vc6	CDC 3525-97	+	US	Clinical
Vc7	CDC 2164-78	+	US	Clinical
Vc8	CDC 3523-03	+	US	Clinical
Vc9	O145B	+	Unknown	Environmental
Vc10	UCIC 305C	+	US – CA	Environmental
Vc11	C-153	+	Peru	Environmental
Vc12	154	+	Unknown	Environmental
Vc13	709-1	+	Unknown	Environmental
Vc14	UCIC 305E	+	US – CA	Environmental
Vc15	CDC 2463-88	+	US	Clinical
Vc16	569B	+	Unknown	Clinical
Vc17	VRL 1984	+	US	Clinical
Vc18	1800-62	+	Unknown	Clinical
Vc19	C-6707	+	Peru	Clinical
Vc21	25-16	+	US - FL	Environmental
Vc22	17-17	+	US - FL	Environmental
Vc23	ATCC 14103	+	India	Clinical
Vc28	D1042	+	Unknown	Environmental
Vc36	G12R	+	Unknown	Environmental
Vc38	SPRC O138	+	US-FL	Environmental
Vc41	INV91-611-863	+	Unknown	Unknown
Vc42	CFSAN 8498	+	Unknown	Environmental
Vc44	SPRC HC5C	+	Unknown	Environmental
Vc46	DAL 315	+	Unknown	Environmental
Vc48	135-17	+	US – FL	Environmental

Table S1 (continued). Results of inclusivity testing for *Vibrio cholerae* isolates using the primers and probes for the *omp*W gene.

Vibrio cholerae				
**FDA GCSL Isolate Number**	**Isolate Name**	**PCR results**	**Source**	**Origin**
Vc50	FRW3 H3	+	AL	Environmental
Vc51	FRO3 B4	+	AL	Environmental
Vc53	FRO3 D4	+	AL	Environmental
Vc54	FRO3 F4	+	AL	Environmental
Vc55	FRO3 G4	+	AL	Environmental
Vc56	FRO3 H4	+	AL	Environmental
Vc57	FRO3 A5	+	AL	Environmental
Vc58	FRO3 B5	+	AL	Environmental
Vc59	FRO3 C5	+	AL	Environmental
Vc60	FRO3 A7	+	AL	Environmental
Vc61	FRO3 B7	+	AL	Environmental
Vc63	FRO3 D7	+	AL	Environmental
Vc64	FRO3 E7	+	AL	Environmental
Vc65	FRO3 F7	+	AL	Environmental
Vc66	FRO3 G7	+	AL	Environmental
Vc67	FRO3 H7	+	AL	Environmental
Vm3	85	+	Unknown	Unknown

Table S2 Results of exclusivity testing for non-vibrio and non-*V. cholerae* isolates using the primers and probes for the *ompW* gene.

Organism	FDA GCSL Isolate Number	Isolate Name	PCR results	Source	Origin
V. vulnificus	Vv1	99-624 DP-C10	(-)	TX	Oyster
	Vv2	99-779 DP-D2	(-)	LA	Oyster
	Vv9	99-743 DP-B6	(-)	TX	Oyster
	Vv10	98-783 DP-A1	(-)	LA	Oyster
	Vv21	ATL 9579	(-)	TX	Clinical
	Vv24	ATL 71504 (CDC 9076-96)	(-)	LA	Clinical
	Vv26	FLA 8869 (CDC 9053-96)	(-)	TX	Clinical
	Vv30	NSV 5736 (CDC 9349-95)	(-)	AL	Clinical
V. parahaemolyticus	Vp1	AN-2416	(-)	Unknown	Unknown
	Vp11	2228-1	(-)	China	Environmental
	Vp21	029-1(b)	(-)	Unknown	Unknown
	Vp33	VP89	(-)	China	Clinical
	Vp42	F11-3A	(-)	WA	Environmental
	Vp43	DI-E12 5/26	(-)	AL	Clinical
	Vp46	SPRC 10295	(-)	WA	Clinical
	Vp55	SAK11	(-)	Japan	Clinical
V. fluvialis	Vf1	DAL 197	(-)	Bangladesh	Clinical
	Vf2	DAL 506	(-)	Unknown	Unknown
	Vf5	DAL 1825	(-)	Unknown	Unknown
	Vf6	DAL 1678	(-)	Unknown	Unknown
V. metschnikovii	Vm1	2908-8	(-)	Unknown	Unknown
V. mimicus	Vm4	85	(-)	Unknown	Unknown
	Vm5	291	(-)	Unknown	Unknown
V. hollisae	Vh1	89A 1960	(-)	Unknown	Unknown
V. alginolyticus	Va30	2208-1B	(-)	AK – US	Environmental
Citrobacter freundii	Nv4	ATCC 8090	(-)	IA - US	Environmental
Citrobacter amalonaticus	Nv5	Hw-3.3	(-)	Unknown	Unknown

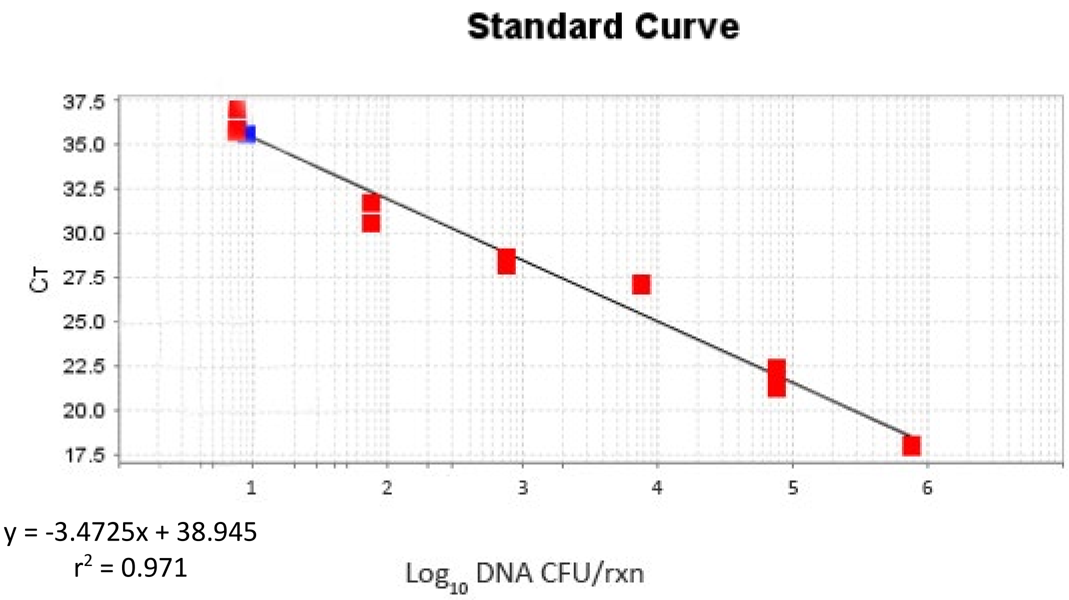

Figure S1 The linear correlation between the C_t values and amount of DNA per mL (Log_{10}), based on CFU/rxn. Limit of detection occurred at the 10^{-6} dilution, which was calculated to contain 0.8 CFU/rxn. Reaction efficiency % = 94.082

References

1. Blackstone, G.M., et al., *Use of a real time PCR assay for detection of the ctxA gene of Vibrio cholerae in an environmental survey of Mobile Bay.* Journal of Microbiological Methods, 2007. **68**(2): p. 254-259.
